# Effects of seining effort on estimates of fish diversity in a sand-bed river

**DOI:** 10.1007/s10661-023-11166-0

**Published:** 2023-04-04

**Authors:** Thomas P. Archdeacon, Eric J. Gonzales, Justin K. Reale, Eileen B. Henry, Joshua D. Grant

**Affiliations:** 1 US Fish & Wildlife Service, New Mexico Fish & Wildlife Conservation Office, Albuquerque, NM 87109 USA; 2 US Bureau of Reclamation, Albuquerque Area Office, Environment & Lands Division, Albuquerque, NM 87102 USA; 3 Environmental Engineering Section, US Army Corps of Engineers, Albuquerque, NM 87109 USA; 4 Present Address: US Forest Service, Lolo National Forest, Ninemile Ranger District, Huson, MT 59846 USA; 5 Present address: New Mexico Department of Game and Fish, Fisheries Management Division, New Mexico, NM 88011 Las Cruces, USA

**Keywords:** Alpha, Beta, Gamma, Community, Assemblage, Stream

## Abstract

Changes in species diversity can be an indicator of ecosystem disturbance, impairment, or recovery. Estimating sampling effort needed to adequately represent stream fish assemblages is necessary for informing conservation actions. Increased sampling intensity can increase species detection, affecting the accuracy and precision of biodiversity indices. Seining is commonly used in fish surveys in sand-bottomed streams of the western USA. Here, we sampled 20, 200-m long stream sites each with 40 consecutive seine hauls to determine how increased within-site effort affected measures of species diversity. An average of 10 seine hauls were required to collect 75% of species present at sites in 40 seine hauls, while 18 seine hauls were required to collect 100% of species observed at a site sampled with 40 hauls. Simpson’s diversity index was highly variable when fewer than 7 seine hauls were performed at each site but stabilized when effort was > 15 seine hauls per site. Total dissimilarity and *β*-diversity components were variable under low sampling effort and also stabilized when effort reached 15 seine hauls per site. However, sampling with more than 18–20 seine hauls per site yielded few additional species. In shallow, sand-bed streams, we suggest sampling with < 5 seine hauls per 200 m of stream can result in unreliable estimates of *α*-diversity and variation in *β*-diversity. Increased effort of 15–20 seine hauls per 200 m of stream captured nearly all species present in 40 hauls per 200 m and stabilized species evenness and *β*-diversity indices.

## Introduction

Species diversity or the presence of rare species is often used as an indicator of ecosystem impairment in riverine systems (Karr, 1981; Miller et al., 1988; Vadas et al., 2022). Species diversity is generally separated into three categories: *α*-diversity, the site or local-level diversity; γ-diversity, the regional or landscape-level diversity; and *β*-diversity, the rate of change of diversity along a gradient (Tuomisto, 2010; Whittaker, 1972). Estimates of *α*- and γ-diversity are important components of longitudinal studies on temporal or spatial changes in riverine ecosystems or impact analyses (Li et al., 2018). Estimates of *β*-diversity are an important component of community ecology, informing the relationship between local and regional diversity and advising conservation planning (Fontana et al., 2020).

Fish faunas in the sand-bed rivers of the western United States are changing, with many endemic species declining (Hoagstrom et al., 2011). Determining the factors driving change in fish faunas in sand-bed rivers is imperative to implement effective conservation actions. Indices of species diversity are important for determining factors driving fish assemblage structures (Wu et al., 2022; Zbinden & Matthews, 2017). Understanding what factors drive change in fish assemblages through time and space, as well as identifying local areas of high diversity, will be critical for maintaining γ-diversity. Answering these questions requires well-designed monitoring plans, especially when there are multiple objectives for monitoring (Xu et al., 2015).

Defining the scale at which an ecological investigation is implemented is important for both design and inference. Many studies have determined sampling effort needed to adequately represent the diversity of riverine fish assemblages, mostly through electrofishing in non-wadable waters at regional or national scales (Hughes et al., 2021). However, these and other studies are generally aimed at estimating γ-diversity as opposed to *α*-diversity (Angermeier & Smogor, 1995). Following Wiens (1989), these studies held the extent of the investigation constant and increase the number of grains (sampling sites) to determine sampling effort. However, increased grain resolution (within-site sampling intensity) can increase detection probability, in turn affecting the accuracy and precision of these biodiversity indices, patterns of rarity, and estimates of functional diversity (Cao et al., 2002; Pritt & Frimpong, 2014; Zhang et al., 2022). Under sampling can presumably lead to biased estimates of biodiversity indices (Beck et al., 2013) and may even affect patterns of *β*-diversity (Marathe et al., 2021). Increasing grain resolution is also inexpensive compared to increasing the number of grains. However, increasing grain resolution provides diminishing returns on reducing variability of species diversity measures, and effort might be better allocated to increasing the number of grains.

While electrofishing methods are generally more effective and often preferred to seining when examining stream fish diversity (Mercado-Silva & Escandón-Sandoval, 2008; Poos et al., 2007; Wiley & Tsai, 1983), electrofishing is not deployable in all situations. Water quality may negatively affect electrofishing capture efficiency (Price & Peterson, 2010). Electrofishing may also be harmful to fishes, particularly during sensitive life stages (Miranda & Kidwell, 2010; Snyder, 2003; Stewart & Lutnesky, 2014) or may be prohibited for cultural reasons. Seines are an inexpensive, easy to deploy alternative to electrofishing. Seining is appropriate for shallow streams with little habitat complexity, targeting small-bodied fishes (Rabeni et al., 2009).

Seining is commonly used in fisheries investigations in the sand-bottomed streams of the Great Plains of the western United States. Previous studies have evaluated effort needed to estimate species richness in smaller (2–11 m wetted width) Great Plains streams, finding a 200-m river segment unit was needed to capture 90% of species present (Patton et al., 2000). In that study, the seine often spanned the entire stream width. In larger wadable streams, each seine haul is a sub-sample of the entire site, and the total area sampled may be far less than the total wetted area at a site. These seine hauls are aggregated to a site-level measure of catch or species richness to make comparisons to other sites or periods of time (e.g. Hoagstrom et al., 2008; Mullen et al., 2011). However, under sampling within a site can lead to variable and imprecise estimates of species-specific catch rates (Archdeacon et al., 2020). Increasing the grain resolution (number of seine hauls within a site) should provide more robust measures of diversity because the ratio of sampled to unsampled areas is increased.

Developing monitoring programs that produce reliable data will be crucial for combating the effects of climate change and monitoring progress towards recovery of aquatic ecosystems (Radinger et al., 2019). Adequate sampling within sites is important for making valid conservation decisions while balancing resources. Our objectives were to examine how variation in seining effort within a site affected estimates of species biodiversity components. Here, we examined species richness at two spatial scales, reach and site. In both cases, we held the extent and number of grains constant, and manipulated grain resolution to observe changes in diversity estimates at the reach and site scales. Specifically, we used fish assemblage data collected in a sand-bed river and compare changes in *α*, *β*, and Simpson’s diversity index as within-site effort increases, but total sampling sites remains fixed. We expand on the work of Patton et al. (2000) to examine at seining effort needed to estimate *α*-, *β*-, and γ-diversity in a large, wadable, sand-bed river.

## Methods

### Study area

**Fig. 1:**
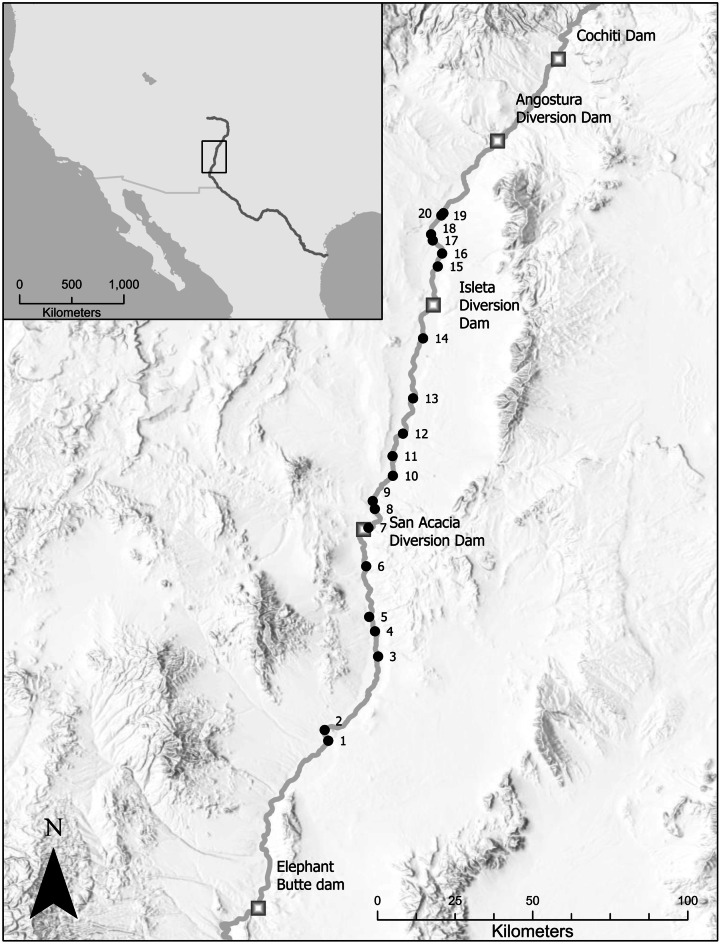
Location of fish sampling sites in the Middle Rio Grande, New Mexico

The Rio Grande is the fifth longest river in the USA, draining an area > 550,000 km^2^ in the USA and Mexico (Patiño-Gomez et al., 2007). For this study, the Middle Rio Grande (MRG) is defined as the section from Cochiti Dam to Elephant Butte Reservoir (Fig. 1). There are no perennial tributaries in this reach. The native fish fauna in the MRG is relatively depauperate (< 20 species) and falls within a single ichthyofaunal zone (Archdeacon et al., 2015; Hoagstrom et al., 2010; McGarvey, 2011). The river is primarily a braided channel sand-bed for much of the MRG (Massong & Tashjian, 2006). We used a generalized random tessellation stratified sampling method to select 20 sites within the MRG. Generalized random tessellation stratified sampling is used to draw spatially balanced sampling locations from the entire target region (Brown et al., 2015). When random sites were selected on tribal lands or no surface water was present when visited, we replaced them with overdraw sites.

The flow regime of the MRG is heavily dependent on snowmelt runoff with high flows in spring, declining to low flows during summer and autumn. The annual flow regime is highly modified, primarily due to water abstractions (Blythe & Schmidt, 2018). Local flow regimes during summer months can vary spatially for a given date, due to upstream water storage, water diversions, irrigation returns, and localized rainfall. Generally, upstream areas of the river channel remain wetted, while downstream areas tend to have substantial areas of intermittent or completely desiccated conditions but may exhibit mid-reach drying patterns on smaller scales due to irrigation return flows and localized precipitation (Archdeacon, 2016).

### Fish collection

Fish assemblage data were collected from 26 to 29 September 2016. Each sampling site consisted of a 200-m section of stream. Sites were sampled upstream to downstream with a small beach seine, 1.0 × 3.0 m, 3.2-mm mesh size. Two people drew the seine downstream through a single mesohabitat of uniform depth and velocity, rapidly lifting the seine out of the water or drawing the seine up a stream bank when present. The length of each haul was measured to estimate effort (nearest 0.1 m); however, hauls were generally 5–12 m in length (12–30 m^2^ per seine haul). The average area sampled per site was 749 m^2^ (SD = 114 m^2^). We sampled each site with 40 seine hauls representing the suite of available mesohabitats. The first 20 seine hauls were performed in all available habitats including proportionally rare habitats (e.g., riffles, pools, and backwaters); hauls 21–40 were in available habitat that did not overlap any of the first 20 seine hauls (generally run habitats). We identified and counted all fish captured in each seine haul. Seine hauls were spaced a minimum of 5 m apart to avoid recapture of the same individuals. After identification, fish were returned to the water alive.

### Data analysis

We chose local species richness (*α*-diversity), local Simpson’s diversity index (evenness), the Bray–Curtis dissimilarity (*β*-diversity), and species richness in the entire study area (γ-diversity) as our indicators of species diversity. At the site level, we calculated the *α*-diversity as observed species richness (*S*_obs_) and compared this to the abundance-based bias-corrected Chao estimator (Colwell & Coddington, 1994; O’Hara, 2005) as seining effort accumulated within a site. Similarly, we calculated the Simpson diversity index as seining effort accumulated within a site. Simpson’s diversity index values near 1 indicate diverse assemblages, whereas values near 0 indicate low diversity. We chose not to randomize seine haul order (e.g., “collector order”) because most of the rare habitats were sampled first, while later seine hauls were relegated to mid-channel run habitats. Using the collector order allowed us to examine how sites would realistically be sampled. We used packages vegan (Oksanen et al., 2020) in program R (R Core Team, 2021) to calculate *S*_Chao_ and Simpson diversity index.

Next, we predicted sampling effort required based on site-level covariates. First, we determined the number of seine hauls required (effort) to collect 75% or 100% of the *S*_obs_ in all 40 aggregated seine hauls within a site. We used three simple linear regression models to relate effort to wetted width (1 m), average depth of seine hauls (0.01 m), and proportion of seine hauls in run habitat. We chose these covariates to represent the size of the sampling location (wetted width), difficulty in sampling with a seine (average seine haul depth), and a coarse measure of habitat heterogeneity (proportion run habitat). We used package DHARMa (Hartig, 2022) to examine residuals of each model and noted no overdispersion, deviance from normality, or outliers.

We used the multiple-site extension of the Bray–Curtis dissimilarity based on species abundance to estimate *β*-diversity (Baselga, 2017). Total dissimilarity (*d*_tot_) can be partitioned into (1) balanced variation (*d*_bal_), where individuals of a species are substituted by equal numbers of a different species at another site (analogous to turnover in presence-absence based patterns), and (2) abundance gradient (*d*_gra_), where individuals are lost from one site to another without replacement (analogous to nestedness in the presence-absence-based patterns). Like *α*-diversity, we calculated the total dissimilarity, partitioned into balanced variation and abundance gradient components, for each cumulative seine haul at all 20 sites. We used package betapart (Baselga et al., 2022) in program R (R Core Team, 2021) to calculate *β*-diversity components.

## Results and discussion

**Fig. 2:**
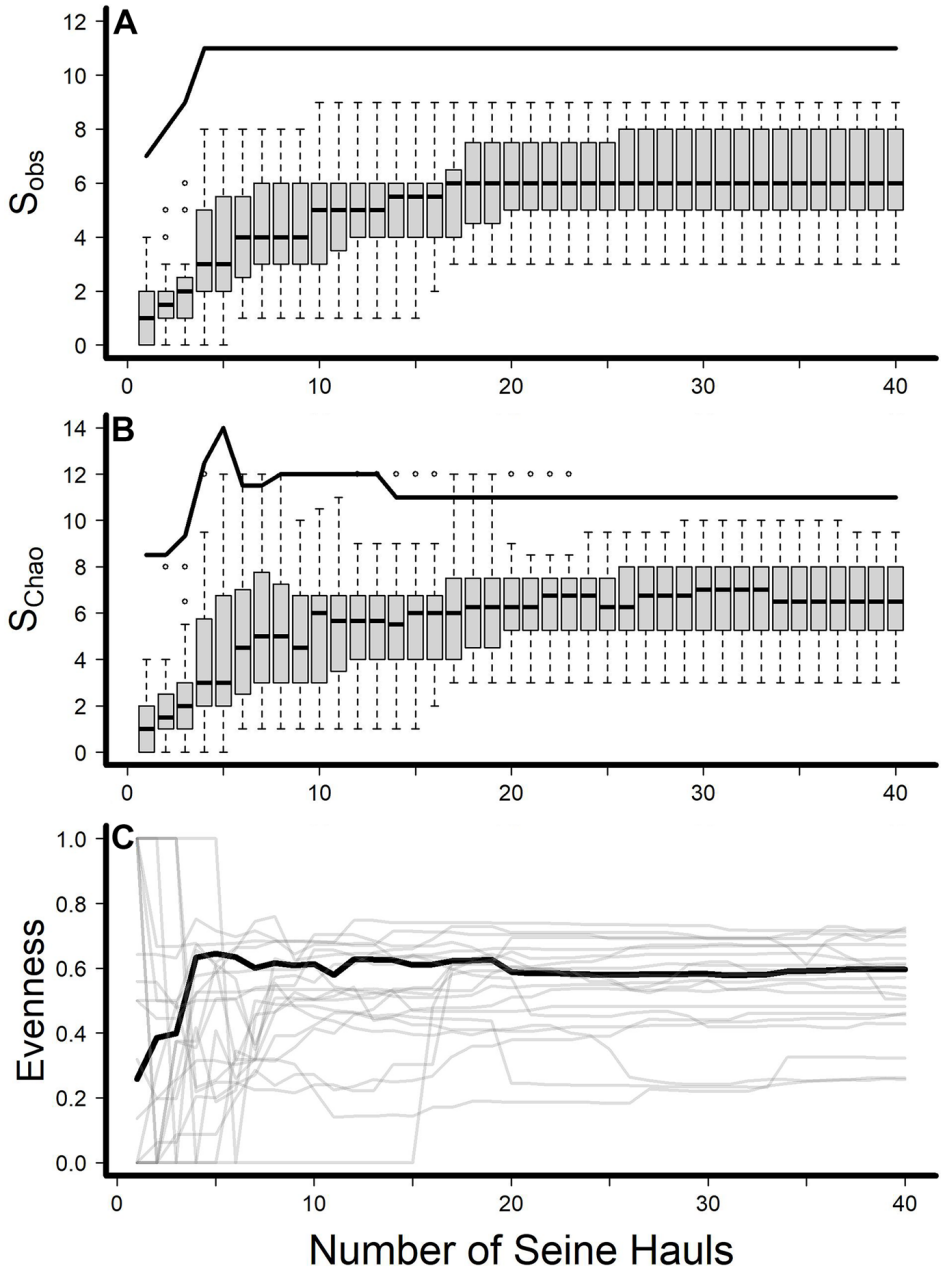
Changes in observed fish species richness (S_obs_), estimated species richness (S_Chao_), and Simpson’s index of assemblage evenness at 20 sites in the Middle Rio Grande, New Mexico, as sites were sampled with increasing numbers of seine hauls. Thick black lines represent observed or estimated γ-diversity **A** and **B **or the overall Simpson’s index from all sites aggregated **C**; thin gray lines represent the individual sites **C**

As expected, species richness accumulated with increasing effort in a site. Among individual sites, *S*_obs_ ranged from 3 to 9 for all 40 seine hauls (Fig. 2A). An average of 10 seine hauls (range 3–20) were required to collect 75% of species present in all 40 seine hauls, while an average of 18 seine hauls (range 3–37) were required to collect 100% of species observed at a site (Table 1). Estimated *α*-diversity (*S*_Chao_) increased similarly (Fig. 2B) and ranged from 3 to 9.5 (Table 1). Observed γ-diversity of 11 species was achieved with only four seine hauls per site; estimated γ-diversity peaked at 14 species with five seine hauls per site but stabilized at 11 species at 14 seine hauls per site (Fig. 2B). Simpson’s diversity index was highly variable when fewer than 7 seine hauls were performed at each site and began to stabilize around 15 seine hauls (Fig. 2C). However, a few sites had variable Simpson’s index values when effort was > 25 seine hauls. Within the study area, observed γ-diversity was 11 species, and the estimated γ-diversity was also 11 species. The overall Simpson’s index for the MRG stabilized at four seine hauls per site (Fig. 2C). Regression analyses revealed no significant predictors among those tested for effort required to reach 75% or 100% of observed species at a site (Table 2).

**Table 1:** Number of fish species captured in the Middle Rio Grande, New Mexico, with 5 (*S*_5_), 10 (*S*_10_), 20 (*S*_20_), and 40 (*S*_40_) seine hauls per site, the Chao species-richness estimator based on all 40 hauls, the number of hauls needed to reach 75% or 100% of all species observed at a site (*H*_75%_ and *H*_100%_, respectively), the proportion of run habitat sampled, average seine haul depth (m), and the wetted width of the site (m)

Site	*S*_5_	*S*_10_	*S*_20_	*S*_40_	Chao	Hauls_75%_	Hauls_100%_	Run	Depth	Width
1	1	6	6	6	7	10	10	0.78	0.17	15
2	3	3	4	4	4	5	12	0.58	0.18	20
3	1	3	6	6	6	16	17	0.67	0.16	9
4	4	5	6	6	6	10	14	0.68	0.18	15
5	3	6	6	8	8.5	7	39	0.41	0.19	10
6	3	3	4	5	5	19	22	0.61	0.18	13
7	0	1	4	4	4	17	20	1.00	0.54	28
8	5	6	6	6	6	5	7	0.48	0.37	30
9	4	5	5	5	5	4	10	0.90	0.40	60
10	3	4	5	5	6	7	18	0.66	0.23	5
11	2	2	3	3	3	16	16	0.76	0.18	9
12	2	3	5	5	5.5	14	20	0.52	0.18	12
13	6	6	6	6	7	3	3	0.55	0.18	3
14	6	8	8	8	8	4	8	0.55	0.17	13
15	8	9	9	9	9	4	10	0.96	0.38	60
16	6	6	8	8	8	4	17	0.78	0.24	100
17	5	5	8	8	8	17	18	0.85	0.37	80
18	2	4	6	7	7.5	20	27	0.90	0.36	40
19	3	8	8	9	9	8	37	0.90	0.27	80
20	6	7	7	9	9.5	9	30	0.83	0.37	60
Average	3.7	5	6	6.4	6.6	10	18	0.72	0.27	33

**Table 2:** Parameter estimates relating the wetted width, average seine haul depth, and proportion of run habitat sampled to effort (number of seine hauls) required to observe 75% (*H*_75%_) or 100% (*H*_100%_) of fish species present in 40 hauls at 20 sites sampled in the Middle Rio Grande, New Mexico

Model	Parameter	Estimate	*R*^2^	*P* value
*H*_75%_				
	Wetted width	−0.03 (0.05)	−0.03	0.54
	Depth	5.60 (12.4)	−0.04	0.66
	Run	8.94 (7.64)	0.02	0.26
*H*_100%_				
	Wetted width	0.07 (0.08)	−0.01	0.37
	Depth	7.0 (20.6)	−0.05	0.74
	Run	6.9 (13.1)	−0.04	0.61

**Fig. 3:**
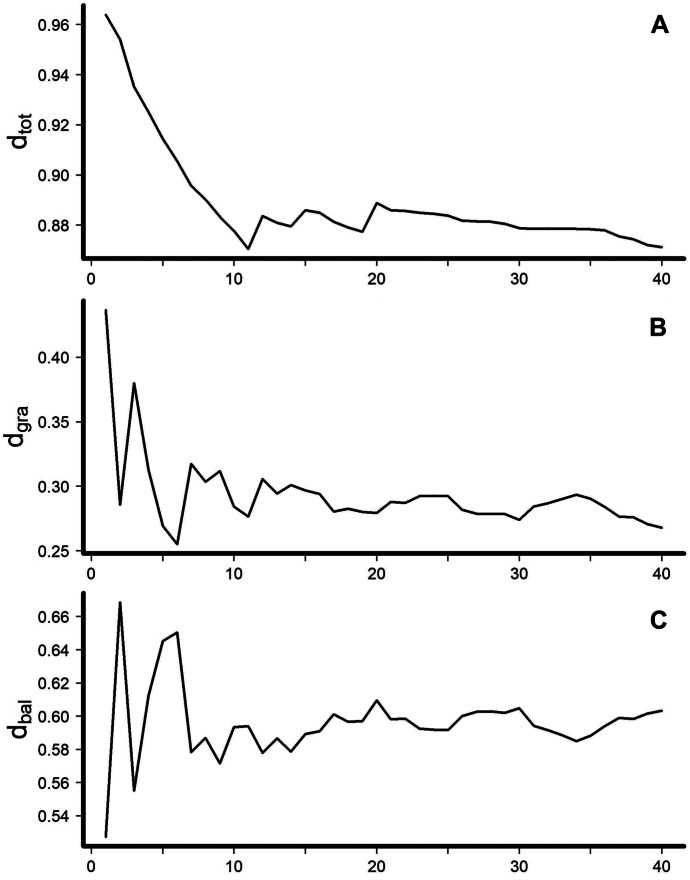
Changes in *β*-diversity of fish assemblage species richness including the Bray–Curtis total dissimilarity (*d*_tot_), abundance gradient component (*d*_gra_), and balanced variation component (*d*_bal_) at 20 sites in the Middle Rio Grande, New Mexico, as sites were sampled with increasing numbers of seine hauls

Total dissimilarity and *β*-diversity components were variable under low sampling effort (Fig. 3). Total dissimilarity declined from 0.96 stabilizing around 0.88 after 10 seine hauls per site (Fig. 3A). The abundance gradient* β*-diversity component was unstable when sites were sampled with < 10 seine hauls, composing approximately 0.25 to 0.44 of total dissimilarity before stabilizing around 0.28 after 15 seine hauls per site (Fig. 3B). Similarly, the balanced variation *β*-diversity component was unstable when sites were sampled with < 10 seine hauls, varying from 0.52 to 0.67 before stabilizing around 0.60 after 15 seine hauls per site (Fig. 3C).

Comparisons among *α*- and γ-diversity and species evenness under differing sampling efforts highlight the importance of aligning monitoring objectives with sampling design. As expected, *α*-diversity increased with increasing effort. However, sampling with more than 18–20 seine hauls per site yielded few additional species. Only five of the 20 sites increased *S*_obs_ when doubling sampling effort from 20 to 40 hauls. Severe under sampling of < 5 seine hauls per site underestimated *α*-diversity by about 50%. This is in stark contrast to γ-diversity (11 species) which required only four seine hauls per site. Similarly, the aggregated MRG-wide Simpson’s index stabilized after five seine hauls per site, far fewer than required for any individual site. These effects on variability of species diversity indices are predicted by Wiens (1989), and it may be more efficient to increase the number of grains, each sampled with lower intensity. However, if observing patterns in *α*-diversity is a monitoring objective, within-site effort is an important consideration as low sampling intensity will severely underestimate *α*-diversity and return evenness indices that are not representative of the specific sampling location.

Comparisons of *β*-diversity and components under varying sampling intensity were more variable than *α*- or γ-diversity. Overall, sites had few species in common, but the differences decreased with increasing grain resolution, a pattern again predicted by Wiens (1989). Even under the highest grain resolution, total dissimilarity was high as sites had varying species abundances. Components of total dissimilarity, which explains how individual sites contribute to γ-diversity, were sensitive to under sampling. At five or fewer seine hauls per site, the abundance gradient component was variable but generally contributed more to total dissimilarity compared to the full 40 seine haul dataset. Conversely, the balanced variation component fluctuated when five or fewer hauls were made per site but did not show a directional trend. Under low within sampling site effort, the relative contributions of each *β*-diversity component appeared relatively equal. With high-resolution grains, it is apparent about 75% of the total dissimilarity is due to balanced variation in abundance, where abundance of one species declines, while another increases from one site to another. Only 25% of the total dissimilarity was due to the abundance gradient component, where abundance decreases from one site to another without replacement.

The variable diversity indices we observed illustrate the importance of adequate within-site sampling. Neither γ-diversity nor regional species evenness were affected by increased within site sampling > 5 seine hauls per site. However, site-specific species evenness, *α*-diversity, and *β*-diversity were significantly affected by under sampling within a site and required > 15 seine hauls per site to stabilize these indices. This has important implications for studies where the sites themselves are of interest. Before-after control-impact studies on fish assemblage response to habitat restoration or catastrophic events (e.g., Reale et al., 2021; Santos et al., 2022) assume sampling efforts sufficient to represent the fish assemblage at a given location and time, allowing observation of real changes in the assemblage. Retrospective analyses from museum collections such as Hughes et al. (2022) should be cautious when sampling effort was not recorded.

Our results can guide future monitoring of stream fish assemblages sampled with seines, particularly in temperate streams with low diversity. Although we found no relation between the sampling condition at a specific site and the amount of effort needed to represent *α*-diversity, application of our results to other river basins will likely need some adjustment. Intuitively, smaller streams may require less effort than 15 hauls per 200 m, as there is greater sampling coverage within a site, but our results did not suggest this relationship. Tropical streams or other highly diverse streams may require considerably more effort to estimate diversity (Pompeu et al., 2021). But, even in highly diverse streams, increasing grain resolution will be met with diminishing returns on variability in diversity estimates.

Our results in the MRG are like studies in other sand-bed systems. In the Pecos River, New Mexico, 10 to 14 seine hauls per site resulted in a fish assemblage similar to more intense depletion sampling methods (Archdeacon & Davenport, 2013). Fish sampling in sand-bed rivers of Oklahoma, USA, suggested that > 10 seine hauls should be performed to increase the probability of detection of rare fishes (Mollenhauer et al., 2018). Taken together, regardless of stream size, sampling 200-m stream sections with > 10 seine hauls results in more accurate representations of stream fish communities in sand-bed rivers.

Properly framing key objectives of monitoring is a critical first step to designing surveys that can collect data to meet those needs (Radinger et al., 2019). The use of multiple gears can improve monitoring efforts for stream fish biodiversity (Zajicek & Wolter, 2018). However, monitoring designs should consider the trade-off of increased labor and less straightforward data analyses, as well as comparability to other studies (Vadas et al., 2022). In shallow, sand-bed streams, increasing within-site effort to 15 seine hauls per site would require a relatively small increase in labor costs, whereas sampling with additional methods may add considerable effort to achieve similar results. In the Middle Rio Grande, increasing within-site effort beyond 20 seine hauls had little effect on our estimates of species diversity, and effort may be better spent surveying a larger suite of locations.

## Conclusions

Within-site under sampling of stream fishes can result in inaccurate assessments of diversity. Estimates of *β*-diversity were particularly influenced by under sampling in our study. Collecting reliable species diversity information is needed for conservation efforts and impact analyses. We suggest the implementation of small-scale studies to determine the amount of within site effort needed for reliable estimates of diversity. For wadeable, shallow, species-poor streams, we suggest sampling with < 5 seine hauls per 200 m of stream is not adequate, underestimating *α*-diversity and causing variation in *β*-diversity. Increasing within site effort to 15–20 seine hauls per 200 m of stream captured nearly all species present in 40 hauls per 200 m and stabilized species evenness and *β*-diversity indices.

## Data Availability

The data used in this study are available at: www.doi.org/10.17632/7rswnkbfyp.1.
